# Biotransformation and kinetics of selected benzimidazole synthetic opioids in human hepatocytes

**DOI:** 10.3389/fphar.2025.1656437

**Published:** 2025-11-27

**Authors:** Gajanan R. Jadhav, Pius S. Fasinu

**Affiliations:** Department of Medical Education, Heersink School of Medicine, The University of Alabama at Birmingham, Birmingham, AL, United States

**Keywords:** benzimidazole opioids, biotransformation, human hepatocytes, metabolism, nitazenes

## Abstract

**Background/Objectives:**

The reemergence of 2-benzylbenzimidazole opioids, also called nitazenes, in the illicit drug market constitutes a serious threat to public health. One of the major challenges in handling exposures and managing intoxications in humans is the poor understanding of the kinetics and biotransformation pathways of these drugs. Although the scheduled status of nitazenes limits interventional clinical studies in humans, liver-based *in vitro* studies can provide insights into their metabolism and pharmacokinetics.

**Methods:**

Three nitazene analogs–butonitazene, isotonitazene, and protonitazene—were incubated in primary human hepatocytes. The depletion rate was profiled against time for metabolic kinetic analysis. Qualitative and quantitative analyses of the incubates were conducted using liquid chromatography–high-resolution tandem mass spectrometry.

**Results:**

All three analogs were rapidly metabolized in hepatocytes, with intrinsic clearance values of 2.4, 3.0, and 3.9 mL/min/g liver for butonitazene, isotonitazene, and protonitazene, respectively, yielding products of multiple metabolic reactions, including hydroxylation, *N*-dealkylation, glucuronidation, and acetylation. The extrapolated *in vivo* clearance [(mL/min)/kg body mass] values of butonitazene, isotonitazene, and protonitazene were 14.4, 15.2, and 16, respectively, compared to 15.5 and 18 for 7-hydroxycoumarin and testosterone, respectively.

**Conclusion:**

Nitazenes are susceptible to hepatic metabolism through hydroxylation, *N*-dealkylation, and conjugation. The extrapolated *in vivo* metabolic clearance is similar to that of 7-hydroxycoumarin and testosterone. For practical purposes, these findings can provide useful estimations in clinical toxicology and forensic pathology.

## 1 Introduction

Nitazenes are potent synthetic opioids with high abuse potential. First synthesized and studied in the 1950s as potential alternative opioid analgesics, nitazenes have become increasingly popular in the illicit drug market following their reemergence in 2019 (Peacock et al., 2019; Rinaldi et al., 2020; Simão et al., 2022). Within this short time, the United States Drug Enforcement Agency (DEA) has identified and categorized at least seven nitazene derivatives as schedule I drugs (United States Drug Enforcement Agency, 2024). Since the discontinuation of their development in the 1950s, few scientific studies have been conducted on nitazenes until their recent reemergence. Thus, information available on this class of drugs is largely from the early discovery studies. The pharmacology, as understood, is based on the strong affinity of the benzimidazole core (due to its planar and electron-rich aromatic structure) for µ-opioid receptor binding (Vandeputte et al., 2024). Modifications to this nitazene moiety, such as alkyl substitutions, can significantly influence the potency, duration of action, and lipophilicity (Glatfelter et al., 2023). Despite these distinct structural properties, nitazenes share similar pharmacodynamic properties with traditional opioids, including potential for high potency and abuse. The pharmacokinetics of nitazenes in humans is not understood, and as their schedule status precludes legal use in humans, the conduct of interventional studies is unfeasible.

Although published reports of intoxications and contaminations with nitazenes have identified multiple metabolites, a wide knowledge gap remains regarding their biotransformation pathways and metabolic stability. For example, the core benzylbenzimidazole structure, which is the pharmacophoric unit for the opioid effect, allows for structural diversification that has led to the identification of dozens of potent nitazenes (Table 1). The paucity of data on the bioconversion of nitazenes could, therefore, make it challenging to distinguish between stand-alone drugs and products of metabolism in toxicological samples. Recent studies have shown the metabolic susceptibility of nitazenes to multiple isoforms of cytochrome P450 (CYP) enzymes. For example, in an *in vitro* incubation in human liver microsomes, protonitazene was metabolized to multiple metabolites, including N-desethylprotonitazene, 5-amino-protonitazene, and 4-hydroxynitazene (Ameline et al., 2024). In another study, butonitazene, isotonitazene, and protonitazene were rapidly depleted when incubated in human liver microsomes and S9 fractions with *in vitro* clearance up to six times that of the control substrates (verapamil and testosterone) (Jadhav and Fasinu, 2024). Both phase 1 and phase 2 metabolic products have been reported with some nitazenes in human hepatocytes (Kanamori et al., 2024). Following the incubation of isotonitazene, metonitazene, etodesnitazene, and metodesnitazene in pooled human hepatocytes, multiple metabolites generated from N-alkylation, O-dealkylation, and glucuronidation were reported (Taoussi et al., 2024). The susceptibility of nitazenes to CYP-catalyzed metabolism, particularly the highly polymorphic CYP2D6 and CYP2C8, also raises concerns about the roles of genetics in predisposition to intoxication and addiction.

**TABLE 1:** Molecular structures of benzimidazole opioids (nitazenes) [reproduced from Jadhav and Fasinu (2024)].

Nitazene	R1	R2	R3	R4
4-Hydroxy nitazenes	NO_2_	OH	CH_2_CH_3_	CH_2_CH_3_
5-Amino isotonitazene	NH2	OCH(CH_3_)_2_	CH_2_CH_3_	CH_2_CH_3_
Butonitazene	NO_2_	OCH_2_CH_2_CH_2_CH_3_	CH_2_CH_3_	CH_2_CH_3_
Clonitazene	NO_2_	Cl	CH_2_CH_3_	CH_2_CH_3_
N-Desethylisonitazene	NO_2_	OCH(CH_3_)_2_	-	CH_2_CH_3_
N-Desetyletonitazene	NO_2_	OCH_2_CH_3_	CH_2_CH_3_	-
Etodesnitazene	H	OCH_2_CH_3_	CH_2_CH_3_	CH_2_CH_3_
Etomethazene	H	OCH_2_CH_3_	CH_2_CH_3_	CH_2_CH_3_
Etoetonitazene	NO_2_	OCH2CH2OCH2CH3	CH_2_CH_3_	CH_2_CH_3_
Etonitazene	NO_2_	OCH_2_CH_3_	CH_2_CH_3_	CH_2_CH_3_
Etonitazepipne	NO_2_	OCH_2_CH_3_	-CH_2_CH_2_CH_2_CH_2_-	
Etonitazepyne	NO_2_	OCH_2_CH_3_	-CH_2_CH_2_CH_2_CH_2_-	
Flunitazene	NO_2_	F	CH_2_CH_3_	CH_2_CH_3_
Isotodesnitazene	H	OCH(CH_3_)_2_	CH_2_CH_3_	CH_2_CH_3_
Isotonitazene	NO_2_	OCH(CH_3_)_2_	CH_2_CH_3_	CH_2_CH_3_
Methylthionitazene	NO_2_	SCH_3_	CH_2_CH_3_	CH_2_CH_3_
Metodesnitazene	H	OCH_3_	CH_2_CH_3_	CH_2_CH_3_
Metonitazene or α-methylmetonitazene	NO_2_	OCH_3_	CH_2_CH_3_	CH_2_CH_3_
N-piperidino etonitazene	NO_2_	OCH2CH3	--CH_2_CH_2_CH_2_CH_2_-	
Propylnitazene	NO_2_	CH_2_CH_2_CH_3_	CH_2_CH_3_	CH_2_CH_3_
Protonitazene	NO_2_	OCH_2_CH_2_CH_3_	CH_2_CH_3_	CH_2_CH_3_

Although the existing studies provide insights into human metabolism of nitazenes, gaps still exist in understanding the time-course and clearance of nitazenes in humans. Therefore, the aim of the current study was to characterize the time-course and biotransformation of three nitazenes—butonitazene, isotonitazene, and protonitazene—in primary human hepatocytes utilizing the analysis of *in vitro* metabolic kinetics for *in vivo* extrapolations.

## 2 Materials and methods

### 2.1 Materials

Cryopreserved primary human hepatocytes (Liverpool® 20-donor, mixed-gender) and hepatocyte culture media (Invitrogro HT and Invitrogro KHB) were procured from BioIVT (Hicksville, NY, United States), whereas stock solutions (1 mg/mL each) of butonitazene, isotonitazene, and protonitazene were purchased from Cayman Chemicals Company (Ann Arbor, MI, United States). Diclofenac, sodium phosphate monobasic, and sodium phosphate dibasic buffers were procured from Sigma-Aldrich (St. Louis, MO, United States), and the analytical columns were procured from Phenomenex (Torrance, CA, United States). HPLC-grade acetonitrile, methanol, and trypan blue were purchased from Thermo Fisher Scientific (Fair Lawn, NJ, United States).

### 2.2 Hepatocyte incubation

The cryopreserved hepatocyte mixture was thawed in water bath (set at 37 °C temperature) and suspended in the prewarmed recovery media. This was followed by centrifugation (300*g*, 5 min) and resuspension of the hepatocyte pellets in the plating media following manufacturer instructions. The resuspended mixture was sampled (at time zero and at predetermined time intervals) for percent viability determination calculated using the trypan blue exclusion method (Vandeputte et al., 2024). The cell suspension was adjusted to approximately 1 million cells per mL count and pipetted into the 12-well plates in aliquots of 500 µL per well. The plates were preincubated for 5 min in a humidified atmosphere (95% air and 5% CO2) in an Eppendorf incubator (Hauppauge, NY, United States) to which a shaker (60 RPM, 37 °C) was attached. Biotransformation reactions were initiated with the addition of the test compounds (nitazenes) or positive control solutions. Test compounds were incubated at an initial concentration of 1 µM. The nitazenes were sourced as pre-formulation solutions in methanol. To prevent the interfering effect of organic solvent on hepatocyte activity, the final concentration of ethanol was maintained at less than 0.5% in all incubations. Both 7-hydroxycoumarin and testosterone were utilized as positive controls to assess the metabolic activity of the hepatocytes under similar incubation conditions of test items. Blank sample (no test item/substrate) incubations were simultaneously performed as negative controls for the metabolite profiling study to rule out any interference from the media or hepatocytes.

Aliquots were sampled at 0, 5, 15, 30, 60, and 120 min to assess for rapid metabolic changes and slow-forming metabolites. The metabolic mixture was then quenched in equal-volume ice-cold methanol. The quenched samples were maintained at −70 °C in a frozen condition until further analysis.

### 2.3 Sample preparation

Samples were thawed at room temperature and vortexed for mixing. Aliquots of 25 µL were collected in another 96-well plate and mixed with 125 µL of methanol–internal standard solution (25 ng/mL diclofenac) for metabolite profiling and metabolic stability assessment. The mixtures were centrifuged at 6 °C (300 *g*, 10 min). Aliquots (100 µL) of the supernatants were transferred to the autosampler vials for LC-MS/MS analysis.

### 2.4 Sample analysis for metabolic stability and metabolite profiling

Samples were analyzed using the previously reported method (Jadhav and Fasinu, 2024). In brief, a simultaneous liquid chromatography–mass spectrometry (LC–MS) method utilizing reversed-phase chromatographic conditions was used. A Phenomenex analytical column (Synergi, Polar RP, 100 A°, 50 * 2.0 mm, 2.5 µ) was utilized as a retention stationary phase for a 7.5-min analytical runtime on ABSciex API 4500 and 5600 Triple-TOF mass spectrometers (for metabolic stability and metabolite identification, respectively), coupled with Schimadzu liquid chromatography. Analytes were eluted in a gradient mode with a flow rate of 250 μL/min using mobile phase systems A and B containing 0.1% formic acid in aqueous (5 mM ammonium formate) and organic (methanol) solvents, respectively. The controller was started at 0.01 min at 40% pump B concentration, maintained for 1 min, decreased to 30% for another minute, increased to 60% until 5.5 min, and then decreased to 40% in the next 1 minute. A solution of diclofenac (25 ng/mL) was used as an internal standard.

For the metabolite profiling study in Analyst software (ABSciex, Toronto, Canada), the stationary phases were Luna® Omega (1.6 µm, 100, 100 * 2.1 mm) and Phenomenex (Torrance, CA) analytical columns, with elution phases comprising aqueous (0.1% formic acid in water) and organic (0.1% formic acid in acetonitrile) solvents. The samples, post-elution, were analyzed using method parameters for positive and negative modes of ionizations, set at ±80, ±15–35 eV, ±5,000/4,500 V, 30 psi, 20 psi, 25 psi, and 400 °C for the declustering potential, collision energy range, ion spray voltages, Gas 1 value, Gas 2 value, curtain gas, and interface temperature, respectively.

### 2.5 Data analysis

The area ratio (analyte area/internal standard area) was utilized for calculating percent metabolism at each time point compared to the zero-minute area. Values were incorporated into GraphPad Prism software to estimate the half-life and elimination rate constant (k). The intrinsic clearance was calculated from the half-life and elimination rate constant.

The intrinsic clearance data were input in the well-stirred model (WSM) to extrapolate *in vivo* clearance in human using the following equation (Davies and Morris, 1993; Fasinu et al., 2013):

$$\text{CLh},in\;vivo=\left(\text{Qh}*\text{CLh},\mathrm{int}\right)/\;\left(\text{Qh}+\text{CLh},\mathrm{int}\right),$$

where hepatic blood flow (Qh) = 20 mL/min/kg; intrinsic clearance in human hepatocytes (CLh, int) = k/no. of cells per well * cells per gm liver * liver weight (g/kg); cells per gm liver = 99,000,000; and liver weight = 21 g/kg.

## 3 Results

### 3.1 Metabolic kinetics

**FIGURE 1:**
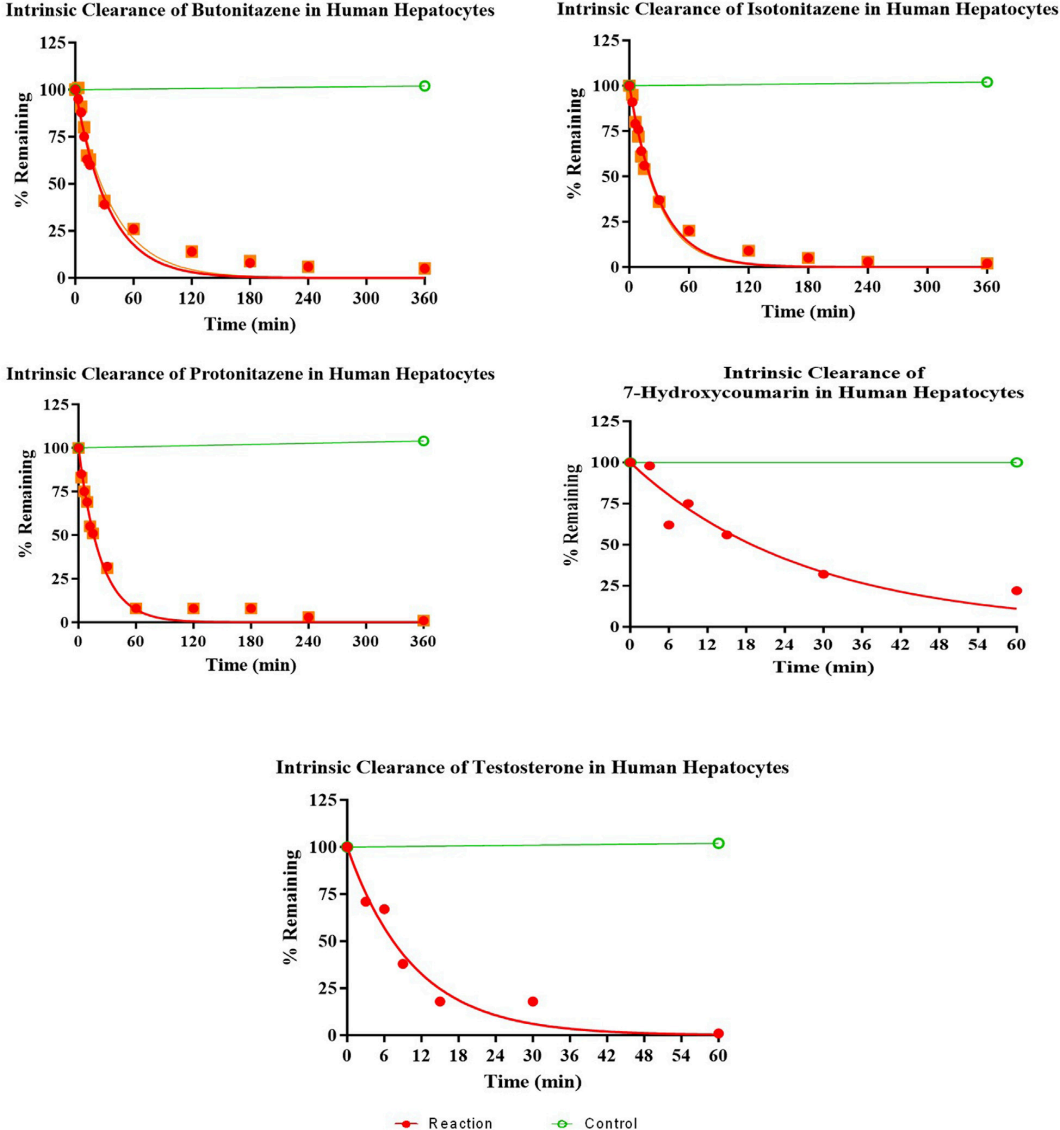
Depletion of nitazenes in human hepatocytes.

The hepatocytes in the incubation mixture had robust viability (>90% viable cells 1-h post-incubation). There was a rapid and extensive metabolism of butonitazene, isotonitazene, and protonitazene by the hepatocytes. The extent of metabolism, although similar, was in the following order: protonitazene > isotonitazene > butonitazene (Table 2; Figure 1). Testosterone and 7-hydroxycoumarin were used as positive controls in this study. Both substrates depend on hepatic clearance in the human body with clearly understood pathways. They have been widely used and recommended as probe substrates for *in vitro* metabolic reactions (Bjornsson et al., 2003; Wang et al., 2005). The metabolic parameters of nitazenes, as observed, were similar to those of the positive controls. The extrapolated *in vivo* clearance [(mL/min)/kg body mass] values of butonitazene, isotonitazene, and protonitazene were 14.4, 15.2, and 16, respectively, compared to 15.5 and 18 for 7-hydroxycoumarin and testosterone, respectively.

**TABLE 2:** Metabolic stability and kinetic parameters of butonitazene, isotonitazene, and protonitazene in human hepatocytes.

Parameter	Butonitazene	Isotonitazene	Protonitazene	7-Hydroxycoumarin	Testosterone
% Metabolism in 60 min	74	80	92	78	99
t1/2 (min)	25	21	16	19	7
CLint (mL/min/g liver)	2.4	3.0	3.9	3.3	8.4
CLint *in vivo* [(mL/min)/kg body mass]	14.4	15.2	16.0	15.5	18.0

% Metabolism, a percentage of substrate depleted over the incubation time with respect to the initial amount at 0 min; t1/2, Time required for depletion of half of the amount of substrate, i.e. 0.963/elimination rate constant (k).

CLint (mL/min/g liver) = k*(vol of reaction/number of cells per m)*cells per gm liver.

CLint *in vivo* [(mL/min)/kg body mass] = [(QH* CLint h)/(QH + CLint H)].

Qh is hepatic blood flow (mL/min/kg), and CLint h is intrinsic clearance in humans = k/cells * (Hepatocell factor/1 g liver weight) * (g of liver weight/kg of body weight).

CLint, intrinsic clearance.

### 3.2 Metabolite identifications and profiling

**FIGURE 2:**
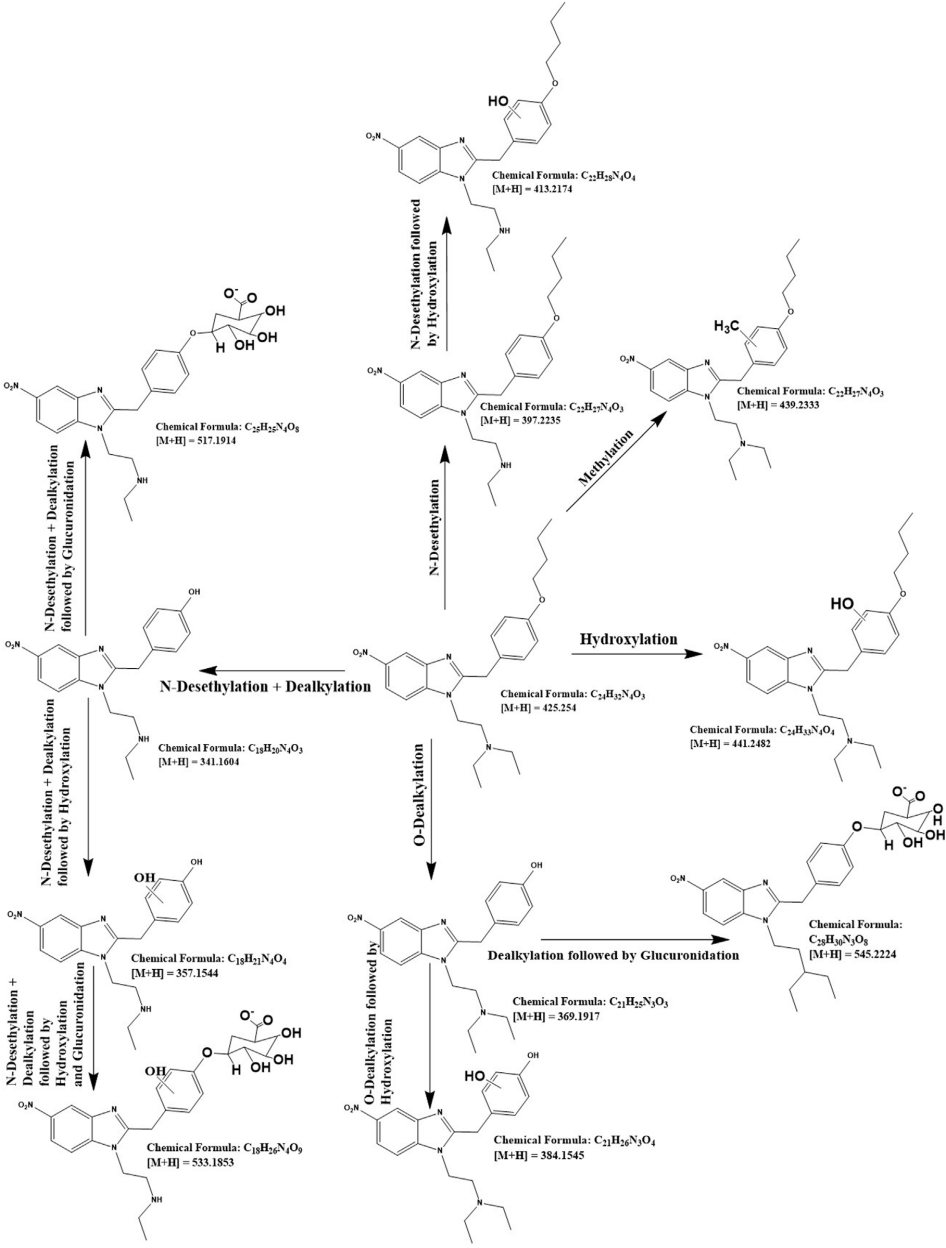
Putative pathway of butonitazene metabolism.

**FIGURE 3:**
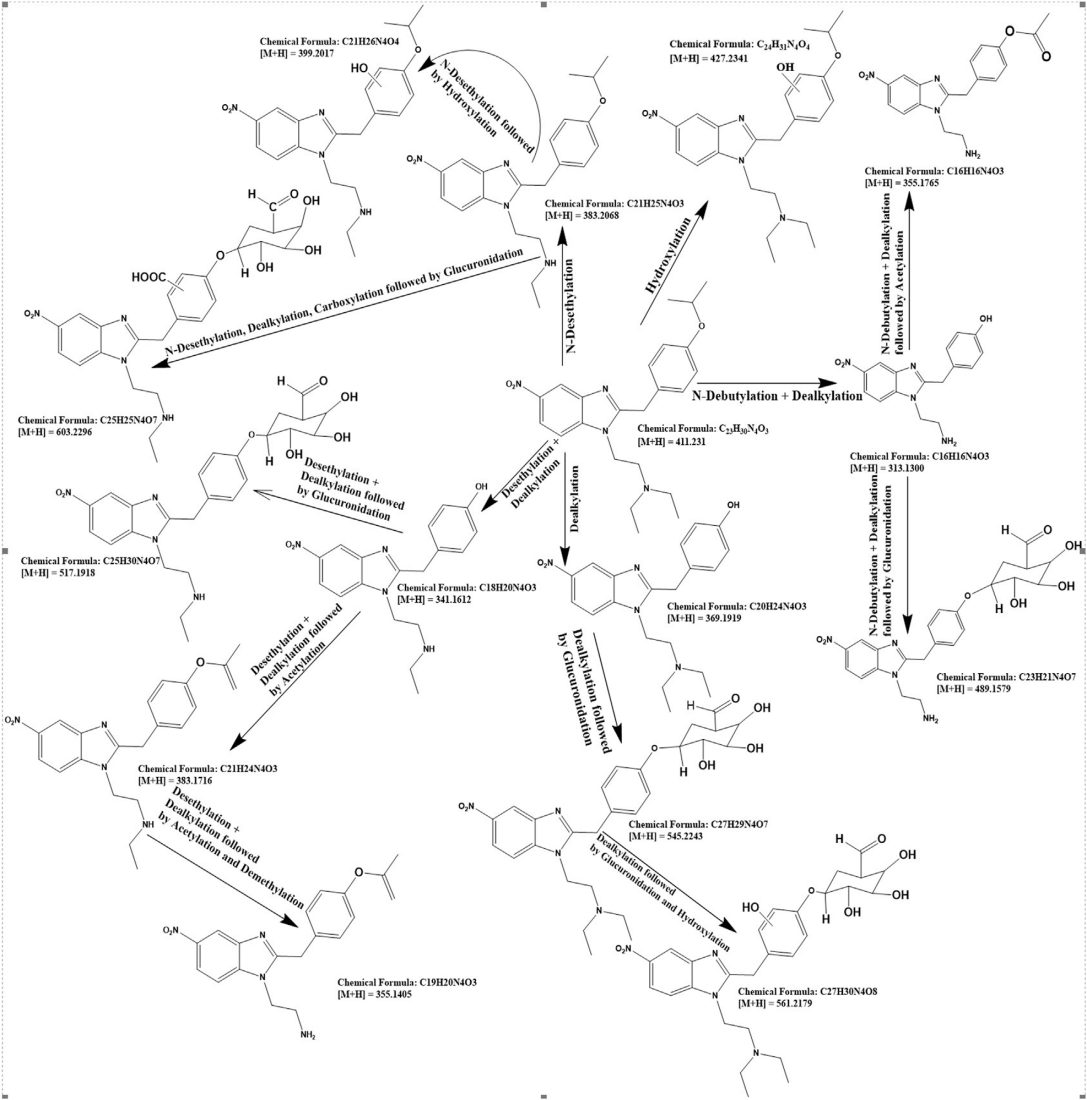
Putative pathway of isotonitazene metabolism.

**FIGURE 4:**
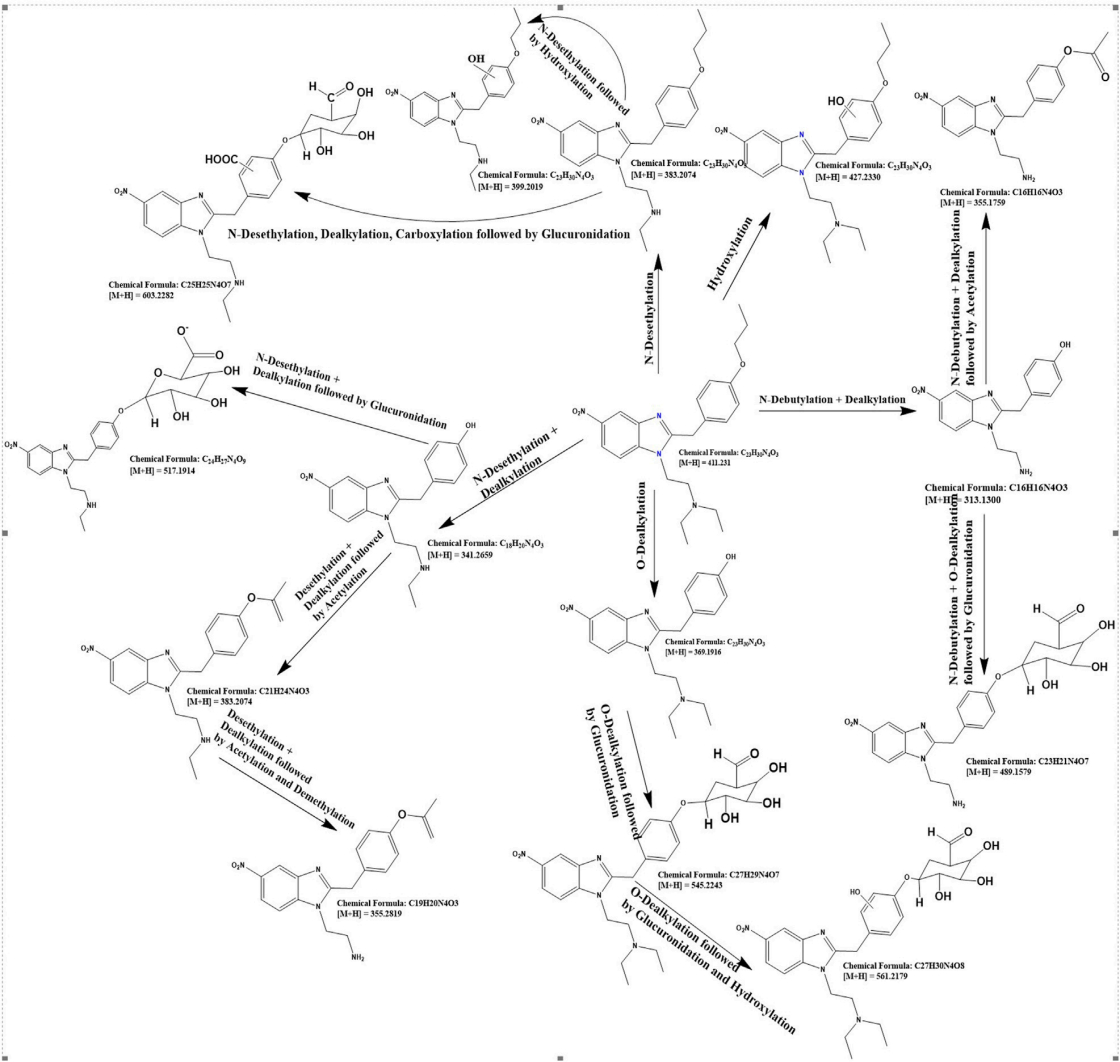
Putative pathway of protonitazene metabolism.

For the detailed understanding of the metabolic characteristics of butonitazene, isotonitazene, and protonitazene, samples were assessed for the presence of putative metabolites or breakdown products in the form of different fragments. The search for metabolites in the accurate mass data was conducted using ABSciex Analyst® software. With reference to previous studies, several biotransformation pathways were evaluated, including, but not limited to, hydroxylation, demethylation, dealkylation, desethylation, dehydrogenation, hydrogenation, dealkylation, glucuronidation, glutathione conjugation, sulfation, acetylation, methylation, glycine conjugation, taurine conjugation, and cysteine conjugation, among others. The acquired data were searched for metabolites using the predicted metabolite mass, mass defects, and fragmentation patterns. Distinction between metabolites and unrelated products was aided using accurate mass comparison. Putative metabolites identified are summarized in Table 3, 4 and Figures 2–4. The primary routes of biotransformation observed for nitazenes include hydroxylation and dealkylation. Putative products of acetylation and glucuronide conjugation were also observed. Based on these products, putative metabolic pathways were proposed for butonitazene, isotonitazene, and protonitazene (Figures 2–4).

**TABLE 3:** Summary of metabolite profiling of nitazenes in human hepatocytes.

Compound	Butonitazene		Isotonitazene		Protonitazene	
	RT	m/z (% Error)	RT	m/z (% Error)	RT	m/z (% Error)
Parent	5.600	425.254 (−0.00024)	4.989	411.231 (−0.00024)	5.178	411.2385 (−0000011)
Hydroxylation	4.243	441.2482 (−0.00041)	3.932	427.2341 (−0.00024)	3.973	427.2330 (0.000154)
N-Desethylation	5.394	397.2235 (−0.00011)	4.790	383.2068 (−0.00024)	5.00	383.2074 (0.0000157)
N-Desethylation, followed by hydroxylation	4.032	413.2174 (−0.00042)	3.538	399.2017 (−0.045100)	3.717	399.2019
N-Desethylation + dealkylation	3.194	341.1604 (−0.00041)	3.207	341.1612 (0.00005181)	3.225	341.2659
N-Desethylation + O-dealkylation, followed by glucuronidation	2.635	517.1914 (−0.00010)	2.612	517.1918 (0.0015789)	2.653	517.1914
N-Desethylation and N-desethylation, followed by O-dealkylation	NA	NA	2.915	313.1300 0.00031)	2.934	313.1300
N-Desethylation and N-desethylation, followed by O-dealkylation and glucuronidation	NA	NA	2.469	489.1579 (0.00001289)	2.485	489.1579
N-Desethylation and N-desethylation, followed by O-dealkylation and acetylation	NA	NA	3.8546	355.131 (0.0001562)	4.738	355.1759
N-Desethylation and N-desethylation, followed by O-dealkylation	NA	NA	4.980	383.1716 (0.002760)	4.971	383.2074
N-Desethylation + O-dealkylation, followed by acetylation and de-ethylation	2.827	533.1853	4.535	355.1405	4.678	355.2819
Dealkylation, followed by glucuronidation	2.856	545.2224	2.866	545.2243 (0.0000171)	2.866	545.2243
Dealkylation + hydroxylation, followed by glucuronidation	NA	NA	2.967	561.2179 (0.00001484)	2.982	561.2179
Dealkylation	5.161	369.1917 (0.00067)	3.491	369.1919 (0.0006589)		369.1916
N-Desethylation + dealkylation + carboxylation + glucuronidation	NA	NA	5.168	603.2296 (0.000000663)	5.359	603.2282
Dealkylation, followed by hydroxylation	4.859	384.1545 (−0.00658)		NA		NA
N-Desethylation + dealkylation, followed by hydroxylation	4.157	357.1544 (0.001232)		NA		NA
Desethylation and dealkylation, followed by hydroxylation and glucuronidation	2.827	533.1853 (0.000061)		NA		NA
Methylation	4.382	439.2333 (−0.00858)		NA		NA

RT, retention time; m/z, mass by charge ratio.

**TABLE 4:** Percent relative abundance of metabolites.

Percent relative abundance							
Butonitazene			Isotonitazene			Protonitazene	
Ion/Fragment	SI	PA	Ion/Fragment	SI	PA	SI	PA
Parent (P1)	-	-	Parent (Iso/Pro-P1)	-	-	-	-
Hydroxylation (Bu-M1)	0.68	0.77	Hydroxylation (Iso or Pro-M1)	0.02	0.04	1.54	1.63
N-Desethylation (Bu-M2)	44	41	N-Desethylation (Iso or Pro-M2)	11.2	13.6	70.50	71.14
Dealkylation (Bu-M3)	4.3	4.39	N-Desethylation, followed by hydroxylation (Iso or Pro-M3)	0.04	0.08	3.32	3.40
N-Desethylation + dealkylation (Bu-M4)	0.97	1.28	N-Desethylation + dealkylation (Iso or Pro-M4)	0.82	1.37	1.30	2.06
N-Desethylation + hydroxylation (Bu-M5)	1.68	2.54	N-Desethylation + dealkylation, followed by glucuronidation (Iso or Pro-M5)	0.22	0.35	0.36	0.43
N-Desethylation + dealkylation, followed by glucuronidation (Bu-M6)	0.22	0.29	N-Debutylation + dealkylation (Iso or Pro-M6)	0.11	0.20	1.30	2.06
Dealkylation, followed by hydroxylation (Bu-M7)	0.22	0.23	N-Debutylation and dealkylation, followed by glucuronidation (Iso or Pro-M7)	0.01	0.01	0.01	0.01
Dealkylation, followed by glucuronidation (Bu-M8)	0.07	0.08	N-Debutylation + dealkylation, followed by acetylation (Iso or Pro-M8)	0.81	1.12	0.01	0.01
N-Desethylation + dealkylation, followed by hydroxylation (Bu-M9)	0.04	0.04	N-Desethylation + dealkylation, followed by acetylation (Iso or Pro-M9)	0.04	0.06	4.98	5.18
Desethylation and dealkylation, followed by glucuronidation (Bu-M10)	0.01	0.02	N-Desethylation + dealkylation, followed by acetylation and demethylation (Iso or Pro-M10)	0.06	0.09	0.07	0.09
Methylation (Bu-M11)	0.40	0.43	Dealkylation, followed by glucuronidation (Iso or Pro-M11)	0.05	0.08	0.07	0.09
-	-	-	Dealkylation + hydroxylation, followed by glucuronidation (Iso or Pro-M12)	0.07	0.10	0.11	0.13
-	-	-	Dealkylation (Iso or Pro-M13)	0.35	0.63	0.72	0.79
-	-	-	N-Desethylation + dealkylation + carboxylation + glucuronidation (Iso or Pro-M14)	0.30	0.38	2.17	2.25

SI, signal intensity; PA, peak area.

## 4 Discussion

The reemergence of nitazenes in the illicit drug market has continued to elicit public health concerns. Apart from their known high-potency opioid effect, the poor understanding of their biotransformation and pharmacokinetics constitutes a major challenge in managing intoxications. Human data on the pharmacokinetics of nitazenes are currently not available as human studies of nitazenes are impractical. The use of human hepatocytes, a metabolic system that provides one of the closest models to the human biotransformation system, offers an alternative to human studies. In our previous study, the metabolic stability of these nitazene compounds was evaluated in human liver microsomes and liver S9 fractions. Reaction phenotyping in recombinant cytochrome P450 enzymes was also conducted (Jadhav and Fasinu, 2024). The study demonstrated the susceptibility of the three nitazenes—butonitazene, isotonitazene, and protonitazene—to microsomal and S9 metabolic activities. Over 90% of the substrates were depleted within 1 hour of incubation, resulting in a half-life of 10 min or less. From the reaction phenotyping study, CYP2D6, CYP2B6, and CYP2C8 were identified as the enzymes responsible for the metabolism of nitazenes (Jadhav and Fasinu, 2024). Although this result provided insights into the metabolic disposition of nitazenes, the *in vivo* predictive capacity of the metabolic systems is generally considered weak.

Primary hepatocytes, being whole-cell, contain the full complement of drug-metabolizing enzymes, including the phase I enzymes (CYP, flavin-containing monooxygenases) and phase II enzymes (including UDP-glucuronosyltransferases, sulfotransferases, and *N*-acetyltransferases). Although cellular subfractions such as microsomes and S9 can provide narrow metabolic profiles, hepatocytes can model complete metabolic pathways, showing products of both phase 1 and 2 reactions, allowing the identification of both intermediate and final metabolites. With known estimates of the number of hepatocytes per liver weight, models exist to extrapolate and upscale *in vitro* results to predict *in vivo* drug clearance. The findings of the current study can, therefore, more closely mirror *in vivo* clearance of butonitazene, isotonitazene, and protonitazene, as modeled in Table 2.

As shown in the results (Figure 1; Table 2), the metabolisms of butonitazene, isotonitazene, and protonitazene were rapid, with almost complete depletion after 2 hours of incubation. The application of the well-stirred model for the extrapolation of clearance values provides insights into human metabolism of these nitazenes. This is important because nitazenes, by virtue of their schedule I status, cannot be objects of interventional studies in humans. There are also no reported *in vitro*–*in vivo* extrapolation (IV–IVE) data for nitazenes. This study, therefore, fills this research gap.

Nitazene metabolic parameters in human hepatocytes are comparative to those of positive controls (7-hydroxycoumarin and testosterone). Although the extrapolated *in vivo* clearance [(mL/min)/kg body mass] values of butonitazene, isotonitazene, and protonitazene are 14.4, 15.2, and 16, respectively, the respective values for 7-hydroxycoumarin and testosterone are 15.5 and 18, respectively. With these comparable extrapolated values, the nitazenes can be expected to demonstrate hepatic clearance similar to that of testosterone in humans. For example, the metabolic clearance of testosterone in humans was reported to be 13L/h/body surface area (Bjornsson et al., 2003). This known value in humans can provide the basis for estimating the clinical metabolic clearance of nitazenes. One limitation to this approach of estimating the clearance of drugs not directly administered in humans is the inability to account for renal contribution to total body clearance.

Typically, 7-hydroxycoumarin mostly get metabolized by phase-II enzymes, including UDPGT and SULT (Wang et al., 2005), whereas testosterone biotransformation is mediated by multiple phase-I (hydroxylation) and phase-II conjugation enzymes, primarily in the liver (Meikle et al., 1987; Li et al., 2018). As mentioned earlier, our previous study demonstrated that butonitazene, isotonitazene, and protonitazene are primarily metabolized by CYP2D6, CYP2B6, and CYP2C8, respectively (Jadhav and Fasinu, 2024). Metabolic studies in hepatocytes, like the current one, provide a more holistic picture of biotransformation, including those from non-CYP and phase 2 enzymes.

The high metabolic clearance observed with nitazenes in this study may also be comparable to other opioids and psychoactive drugs (Constanza Escobar-Wilches et al., 2020). In particular, the contribution of CYP2D6 (the enzymes responsible for the metabolism of most centrally acting drugs) makes nitazene metabolism similar to that of phenanthrene opioids. The observed rapid hepatic clearance can have multiple implications. Drugs that rely on hepatic activity for clearance may be particularly dangerous for individuals with liver diseases. In this population, drug accumulation exacerbates toxicity. Strong affinity for hepatic enzyme activities, as observed with nitazenes, can also contribute to liver toxicity in chronic drug users. For practical forensic and clinical purposes, rapid drug metabolism confounds the detection and identification of the drug of interest in cases of intoxication and abuse. At such instances, it could be difficult to distinguish between the consumed compounds and their metabolic products. This is even more complicated for nitazenes whose metabolites have not yet been fully elucidated.

Some of the metabolites identified in the study have been reported in human sample analysis following acute intoxications with nitazenes (Yanfei Li et al., 2019). Similarly, a study previously conducted in human hepatocytes with metonitazene, etonitazene, and protonitazene reported multiple metabolites, including the products of *N*-desethylation, *N*,*N*-di-desethylation, *O*-desalkylation, *N*-desethyl-*O*-desalkylation, and *N*,*N*-di-desethyl-*O*-desalkylation, along with *N*-oxidated products and *O*-glucuronides of the *O*-dealkylated products (Kanamori et al., 2024). Although some of the current findings are new, the similarity in the metabolite profiles suggests class effects, with strong indication that the benzimidadole opioids are primarily subjected to hepatic metabolism through primary hydroxylation and dealkylation, followed by conjugation reactions. For example, Taoussi et al. (2024) characterized the metabolisms of isotonitazene, metonitazene, etodesnitazene, and metodesnitazene in human hepatocytes. The extent of metabolism and the number of metabolites generated by each of these substrates were different. However, *N*-deethylation at the N,N-diethylethanamine side chain, *O*-dealkylation, and *O*-glucuronidation were common to nitazenes. As demonstrated in the current study with a different set of nitazenes (butonitazene, isotonitazene, and protonitazene), ring hydroxylation, *N*-dealkylation, *O*-dealkylation, and *O*-glucuronidation are recurrent pathways of nitazene biotransformation. The knowledge of the receptor-level activities of these metabolites is of interest for a deeper understanding of the pharmacology of nitazenes. If active, these metabolites may contribute to the known toxicity of nitazenes. The current knowledge of the identities of these metabolites, however, is a useful biomarker in forensic toxicology, drug adulteration, and substance misuse.

## 5 Conclusion

Although direct interventional studies of nitazenes in humans may not be practical, the use of human hepatocytes provides insights into their kinetics and biotransformation. Butonitazene, isotonitazene, and protonitazene were rapidly metabolized in human hepatocytes, yielding products of multiple metabolic reactions, including hydroxylation, *N*-dealkylation, glucuronidation, and acetylation. The results from the use of the well-stirred model suggest that these three nitazenes demonstrate *in vivo* intrinsic metabolic clearance similar to that of 7-hydroxycoumarin and testosterone. This represents the closest metabolic kinetics of nitazenes in humans.

## Data Availability

The original contributions presented in the study are included in the article/Supplementary Material; further inquiries can be directed to the corresponding author.
